# Lack of Cortical Correlates of Response Inhibition in 6-Year-Olds Born Extremely Preterm – Evidence from a Go/NoGo Task in Magnetoencephalographic Recordings

**DOI:** 10.3389/fnhum.2016.00666

**Published:** 2017-01-06

**Authors:** Elina Pihko, Piia Lönnberg, Leena Lauronen, Elina Wolford, Sture Andersson, Aulikki Lano, Marjo Metsäranta, Päivi Nevalainen

**Affiliations:** ^1^Department of Neuroscience and Biomedical Engineering, Aalto UniversityEspoo, Finland; ^2^BioMag Laboratory, HUS Medical Imaging Center, University of Helsinki and Helsinki University HospitalHelsinki, Finland; ^3^Department of Child Neurology, Children’s Hospital, University of Helsinki and Helsinki University HospitalHelsinki, Finland; ^4^Department of Clinical Neurophysiology, Children’s Hospital, HUS Medical Imaging Center, University of Helsinki and Helsinki University HospitalHelsinki, Finland; ^5^Institute of Behavioural Sciences, University of HelsinkiHelsinki, Finland; ^6^Department of Pediatrics, Children’s Hospital, University of Helsinki and Helsinki University HospitalHelsinki, Finland

**Keywords:** children, preterm, magnetoencephalography (MEG), oscillations, response inhibition, somatosensory

## Abstract

Children born extremely preterm (EPT) may have difficulties in response inhibition, but the neural basis of such problems is unknown. We recorded magnetoencephalography (MEG) during a somatosensory Go/NoGo task in 6-year-old children born EPT (*n* = 22) and in children born full term (FT; *n* = 21). The children received tactile stimuli randomly to their left little (target) and index (non-target) finger and were instructed to squeeze a soft toy with the opposite hand every time they felt a stimulus on the little finger. Behaviorally, the EPT children performed worse than the FT children, both in responding to the target finger stimulation and in refraining from responding to the non-target finger stimulation. In MEG, after the non-target finger stimulation (i.e., during the response inhibition), the sensorimotor alpha oscillation levels in the contralateral-to-squeeze hemisphere were elevated in the FT children when compared with a condition with corresponding stimulation but no task (instead the children were listening to a story and not attending to the fingers). This NoGo task effect was absent in the EPT children. Further, in the sensorimotor cortex contralateral to the tactile stimulation, the post-stimulus suppression was less pronounced in the EPT than FT children. We suggest that the missing NoGo task effect and lower suppression of sensorimotor oscillations are markers of deficient functioning of the sensorimotor networks in the EPT children.

## Introduction

Preterm children are at an increased risk of neurodevelopmental problems and long-term cognitive difficulties (Johnson et al., 2009; Kerr-Wilson et al., 2012). Specific areas of weakness for preterm children include attention regulation and executive functions, implicating cognitive functions necessary for achieving goal-directed behavior such as response inhibition, working memory, planning, shifting, and fluency (Mulder et al., 2009). Several studies have shown a negative effect of preterm birth on inhibitory control abilities (Mulder et al., 2011; Orchinik et al., 2011; Aarnoudse-Moens et al., 2012; Baron et al., 2012; Voigt et al., 2012; Loe et al., 2015; Jaekel et al., 2016; Réveillon et al., 2016). Furthermore, self-control abilities—e.g., inhibitory or effortful control—may have a mediatory effect on cognitive outcome (Voigt et al., 2012), attention regulation, and academic achievement (Jaekel et al., 2016). The neural basis of inhibition problems in preterm children is, however, not well understood.

Cortical neural oscillations in the alpha band (8–13 Hz), measured with electroencephalography (EEG) and magnetoencephalography (MEG), have been suggested to have a role in functional inhibition (for reviews see Klimesch et al., 2007; Jensen and Mazaheri, 2010). Prominent alpha-band oscillations sensitive to visual stimulation are present over the occipital areas, and correspondingly, alpha-band oscillations sensitive to tactile stimulation or movement are present over the sensorimotor areas. Suppression of these alpha oscillations, induced by a relevant stimulus, is considered to reflect the initial active information processing in the sense of local cortical excitatory brain processes (see e.g., Pfurtscheller, 1992; Klimesch et al., 2007). The subsequent rebound of the alpha oscillations over the prestimulus level, on the other hand, is suggested to reflect inhibition of the local cortical networks, possibly associated with return of top-down control and readiness to perform a new task (Klimesch et al., 2007). In addition, increased alpha oscillations have been associated with inhibition in other types of tasks as well. In an attention allocation task, increased level of alpha activity suppressed processing of inputs outside the field of attention, while activity in the alpha band decreased in engaged regions (Jensen and Mazaheri, 2010; Händel et al., 2011). Furthermore, increased oscillatory activity in alpha band can be observed in tasks where a learned response must be inhibited (Hummel et al., 2002). Nakata et al. (2013) used MEG to study how sensorimotor oscillations were modulated during a response inhibition task in healthy adults. By applying a sensorimotor Go/NoGo task, they showed that response inhibition after the NoGo stimulation was associated with increased sensorimotor alpha oscillations.

Sensorimotor oscillations are also present in infants and children (Stroganova et al., 1999; Marshall et al., 2002; Berchicci et al., 2011), although they have not been as thoroughly studied as in adults. Here, we set out to study how 6-year-old children born extremely preterm (EPT)—in comparison with children born full term (FT)—perform in a sensorimotor Go/NoGo task requiring response inhibition, and whether their performance is reflected in modulation of the sensorimotor oscillations recorded with MEG. Based on reports of response inhibition problems in preterm children, we hypothesized that the FT children would perform better than the EPT children in the behavioral task. Furthermore, based on results from a similar Go/NoGo study in healthy adults (Nakata et al., 2013), we expected to see elevated oscillation levels in the TASK NoGo condition compared with a condition with the same stimuli but no task (i.e., NO-TASK condition). We further hypothesized the behavioral performance to be reflected in the modulation of the sensorimotor oscillations.

## Materials and Methods

### Participants

The study group comprised 43 6-year-old children of whom 22 were born EPT (gestational age <28 completed weeks) and 21 were born FT (gestational age ≥37 completed weeks). The children were originally recruited for a larger multi-methodological follow-up study (Rahkonen et al., 2013, 2014). The EPT children were born between 2006 and 2008 and treated after birth at the neonatal intensive care unit of the Helsinki University Hospital, Finland. The FT children, including 10 new controls recruited at 6 years of age, were born healthy in the Hospital District of Helsinki and Uusimaa between 2006 and 2009. For this study, we only included EPT children without major brain abnormalities, i.e., grade III–IV intraventricular hemorrhage in cranial ultrasound in the neonatal period or moderate-to-severe white matter abnormality (Woodward et al., 2006) in brain magnetic resonance imaging (MRI) at term age. The included FT children had no known brain abnormalities or neurological disorders.

Altogether, 25 EPT children and 25 FT children underwent the MEG recording. We subsequently excluded three EPT children and four FT children from the present analysis due to an incomplete measurement session (one EPT child and one FT child), extensive movement (two EPT children), technical problems (two FT children), or epileptiform abnormalities in MEG (one FT child). **Table 1** presents details of the clinical characteristics of the 43 participating children included in the final analysis.

**Table 1:** Characteristics of the study groups.

	EPT	FT	EPT vs. FT (*p*)
*N*	22	21	
Gestational age at birth (wk)	26.2 [1.1]	40.4 [0.8]	<0.001^∗^
Birth weight (g)	834 [162]	3640 [337]	<0.001^∗^
SGA	3 (14%)	0	0.2
Boys	12 (55%)	10 (48%)	0.8
Twins	6 (27%)	0	0.02^∗^
BPD	11 (52%)^1^	–	
NEC	0	–	
ROP	7 (32%)	–	
Age in MEG (y)	6.5 [0.1]	6.5 [0.1]	0.5
Left-handed	3 (14%)	1 (5%)	0.6
Full scale IQ	99 [11]^2^	106 [10]	0.02^∗^
Maternal education			0.048^∗^
Low	0	0	
Middle	15 (68%)	8 (38%)	
High	7 (32%)	13 (62%)	

*Data shown as n (%) or mean [SD]. ^∗^p < 0.05 (Independent samples T-test, Fisher’s Exact Test or Chi-square Test according to the variable type and expected frequency within cells.) ^1^Data not available for one EPT child,^2^Data not available for two EPT children. BPD, Bronchopulmonary dysplasia (at gestational age 36 + 0); EPT, extremely preterm; FT, full term; IQ, intelligence quotient; NEC, necrotizing enterocolitis; ROP, retinopathy of prematurity; SGA, small for gestational age (birth weight Z-score < -2 SD)*.

The Ethics Committee for gynaecology and obstetrics, pediatrics and psychiatry of the Hospital District of Helsinki and Uusimaa approved the study protocol. All participants and their parents signed an informed consent in accordance with the Declaration of Helsinki.

### Clinical Data

Obstetric and neonatal data were collected from hospital records and a parental questionnaire. Gestational age was determined from the first-trimester ultrasound when available. Birth weight *z*-scores for gestational age, sex, plurality, and parity were based on the Finnish growth reference data (Sankilampi et al., 2013). General cognitive ability (full scale intelligence quotient) at 6.5 ± 0.1 (mean ± SD) years of age was assessed using five subtests of the Finnish edition of the Wechsler Preschool and Primary Scale of Intelligence – Third Edition, WPPSI-III, (Wechsler et al., 2009). Handedness was determined according to the writing hand. Maternal education was obtained by a parental questionnaire. Educational attainment was classified from low (elementary school) to high (university).

### Measurements

Magnetoencephalography was recorded in the BioMag Laboratory in a magnetically shielded room (Euroshield, Finland) with a helmet-shaped sensor array consisting of 306 independent channels: 204 gradiometers and 102 magnetometers [Elekta Neuromag Vectorview (*n* = 34) or TRIUX (*n* = 9, all FT children), Elekta Oy, Helsinki, Finland]. Only gradiometer data were used in the analysis. The sampling rate was 1004 Hz (TRIUX: 1000 Hz) and the measuring band pass from 0.03 to 251 Hz (TRIUX: 0.03 – 330 Hz). An individual Cartesian coordinate system was constructed for each participant by digitizing the preauricular points and nasion before the recordings. Four (Vectorview) or five (TRIUX) position indicator coils were attached to the head and their positions relative to the anatomical landmarks were digitized. In the beginning of each recording block, the coils were fed with currents to determine the head position relative to the sensors. Continuous head position measurement was applied during the measurement. The activity of the thenar muscles and wrist/finger flexors of the right hand and forearm, respectively, were measured with two bipolar electromyography (EMG) traces.

During the measurement, the child lay supine with his/her head in the measurement helmet. Since the head of a 6-year-old child is small for the adult-size helmet, the head was supported from both sides with thin soft cushions to prevent head movements. During all recording blocks (except for the REST condition with eyes closed), the child was encouraged to look at a colorful picture on the ceiling of the shielded room above his/her head in order to prevent excessive eye-movements. One of the researchers was always in the measurement room with the child to give instructions and ensure optimal behavior. One parent was also allowed to join in.

### Stimulation

Tactile stimulation was applied to the volar side of the distal phalanges of the index and little finger of the left hand. The stimulus was a gentle tap on the skin, delivered by a plastic membrane that expanded with delivered air puffs (Somatosensory Stimulus Generator, 4-D NeuroImaging Inc., San Diego, CA, USA). The stimulation was given in a semi-random predefined sequence (to the index finger with 60% probability and to the little finger with 40% probability) with a 2-s inter-stimulus interval between consecutive stimuli. One block lasted approximately 9 min with at least 250 stimuli.

### Recording Conditions

#### REST Condition

The first block was a resting condition without any stimulation. The child first lay 2 min with eyes open and then 2 min with eyes closed. The data from the eyes-open measurement were used to determine the oscillation frequency over the sensorimotor areas, whereas data from the eyes-closed condition were used to determine the oscillation frequency over the occipital area.

#### TASK Condition

The tactile stimulus was given to the left index and little finger (see ‘Stimulation’ above). The child was instructed to attend to the stimuli and squeeze a small, soft, non-magnetic toy with the opposite, right, hand each time he/she felt the stimulus on the little finger (Go), and to do nothing when he/she felt the stimulus on the index finger (NoGo). The behavioral performance, i.e., the number and correctness of toy-squeezes, was monitored by EMG traces from the right hand and forearm.

#### NO-TASK Condition

The stimulation to the left index and little finger was the same as in the TASK condition, but the child was instructed to ignore the tactile stimulation and instead relax and listen to a story that was read aloud by the researcher in the room. The story was read to make the child comfortable and relaxed and pay no attention to the tactile stimuli.

### Analysis of the MEG Data

The head movement compensation, i.e., the data conversion to the initial head position, as well as the suppression of external magnetic interference from outside the brain, were calculated from the raw MEG data with the spatiotemporal signal space separation method (Taulu and Simola, 2006) in the MaxFilter^®^ software (Elekta Neuromag^®^; Elekta Oy, Helsinki, Finland) using a correlation limit of 0.98 and a 16-s time window. Then, individual spectral content of the data during eyes open and eyes closed was calculated for each subject from the resting data. Furthermore, the child’s head position in the MEG helmet was visualized with the BrainStorm software (Tadel et al., 2011) using an MRI template for 6.5-year-olds (Evans, 2006; Sanchez et al., 2012; Richards and Xie, 2015; Richards et al., 2016). In addition, we calculated the amount of head movements (average translational and rotational speed) in the NO-TASK condition. There were no significant differences in these parameters between the EPT and FT children.

For the TASK condition, EMG traces were inspected visually to identify epochs with correct responses, i.e., squeezing for the little finger stimulation and not squeezing for the index finger stimulation. Only the correct epochs were used in further analyses.

**FIGURE 1:**
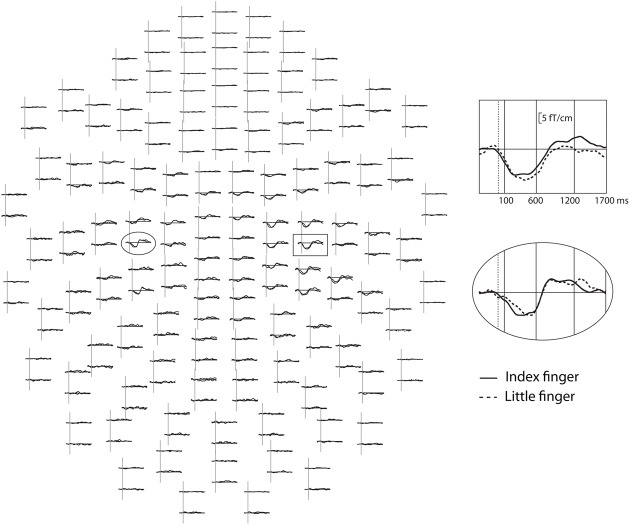
**Modulation of the alpha-band oscillation amplitudes after tactile stimulus to the left hand in the NO-TASK condition in one FT child.** All gradiometer channels are shown as seen from the top of the measurement helmet, nose upward, left hemisphere on the left. In the inserts, one channel from the left (ellipsoid) and right (box) sensorimotor areas. Dotted vertical lines indicate stimulus onset, solid vertical lines show the analysis time-windows, and the solid horizontal lines represent the prestimulus baseline level.

The reactivity of the sensorimotor oscillations in alpha (7–12 Hz) and beta (15–25 Hz) bands was analyzed using the temporal-spectral evolution method (Salmelin and Hari, 1994), where the data are first band-pass filtered, rectified, and finally averaged, time-locked to the tactile stimulation. This demonstrates event-related changes in the average amplitude level of oscillatory activity in the given frequency bands in the same units as the original data. Before calculating the amplitude envelopes, the somatosensory evoked fields (to finger stimulation) were subtracted from the raw data and artifacts from cardiac activity removed by the signal space projection method (Taulu and Simola, 2006). Then, the amplitude envelopes were calculated from all gradiometer channels for each subject in the two frequency ranges, independently for both index and little finger stimulation and for both TASK and NO-TASK conditions (all steps performed using the Graph software, Elekta Neuromag^®^; Elekta Oy, Helsinki, Finland). In the TASK condition, only epochs with correct behavioral responses were included in the averages. Finally, two gradiometer channels in each hemisphere showing the clearest modulation of the oscillation level were averaged and these averages, one in each hemisphere, were used for amplitude comparisons. Averaging two channels was done to add objectivity to the choice, as e.g., suppression and rebound might be maximal in neighboring channels. The amplitude envelopes were then low-pass filtered at 10 Hz. After visual inspection (**Figure 1**), three time windows were chosen for the main analysis: from 100 to 600 ms post-stimulus including the main suppression, from 600 to 1200 ms, and from 1200 to 1700 ms. The mean oscillation amplitude during each of these three time-windows was calculated for each subject and each condition. Relative amplitudes for each time-window were then calculated as percentages of the 300-ms prestimulus baseline. Statistical analysis was performed on these relative amplitudes. In addition, the modulation of occipital alpha-band oscillations was calculated (average of eight occipital channels) from the same three time-windows from epochs of the index-finger stimulation in the TASK and NO-TASK conditions.

The equivalent current dipoles in the primary somatosensory cortex (SI) for the first prominent activity peak after the index finger stimulation in the NO-TASK condition were calculated using a spherical head model with the source modeling program (Elekta Neuromag^®^; Elekta Oy, Helsinki, Finland). Source-level oscillations and their modulations for this SI dipole were then calculated using the Graph program (Elekta Neuromag^®^; Elekta Oy, Helsinki, Finland).

### Statistics

Statistical analyses were performed using the IBM SPSS Statistics software v. 22 (SPSS Inc, Chicago, IL, USA). Baseline characteristics between the study groups were compared with Student’s *T*-test, Fisher’s Exact Test or Chi-square Test according to the variable type and expected frequency within cells. Behavioral results in the Go/NoGo task between and within groups were compared with Mann–Whitney *U*-test and Wilcoxon signed rank test. The correlation between these behavioral results and the intelligence quotient was performed with Spearman’s rho. MEG results were compared with a repeated measures ANOVA (refer to the results section for details of each comparison). Greenhouse-Geisser corrections were used when needed. *P*-values less than 0.05 were considered statistically significant. *Post hoc* comparisons (performed with Student’s *T*-test) were corrected for multiple comparisons with Bonferroni correction when several comparisons were carried out from the same data, e.g., when oscillation levels after index and little finger stimulation were compared in all time-windows separately. In these cases, the original *p*-value was multiplied by the number of comparisons. If this corrected *p*-value was less than 0.05, the difference was considered statistically significant. For the source-level analysis, Student’s *T*-test or, in case of non-normal distribution of the data, Mann–Whitney *U*-Test was used.

## Results

### Behavioral Responses

The EPT children performed worse than the FT children in the TASK condition requiring a squeeze for the little-finger (target) stimulation and no squeeze for the index finger (non-target) stimulation (**Table 2**). In addition, it was more difficult for the EPT children to refrain from squeezing for the non-target finger stimulation than to respond with a squeeze for the target finger stimulation. The FT group had a similar tendency, but the difference between correct response rates for the target and non-target finger was not significant (**Table 2**). Intelligence quotient had no significant correlation with the rate of correct behavioral responses (Spearman’s correlation, *p* = 0.06 for index finger and *p* = 0.14 for little finger).

**Table 2:** Rate of correct behavioral responses [median (range)] to the non-target index finger (NoGo, i.e., no squeeze) and target little finger (Go, i.e., squeeze) stimulation in FT (full term) and EPT (extremely preterm) children.

Correct response rate	FT (*n* = 21)	EPT (*n* = 22)	FT vs. EPT^1^
Index finger (NoGo)	86 (56–95) %	71 (23–92) %	*p* = 0.03^∗^
Little finger (Go)	89 (71–100) %	79 (61–100) %	*p* = 0.049^∗^
Index vs. little^2^	*p* = 0.09	*p* = 0.01^∗^	

*^1^Independent samples Mann–Whitney U-test, corrected; ^2^Related samples Wilcoxon signed rank test, corrected; ^∗^p < 0.05.*

### MEG

#### Reactivity of the Sensorimotor Oscillations

##### NO-TASK condition. Alpha band

**FIGURE 2:**
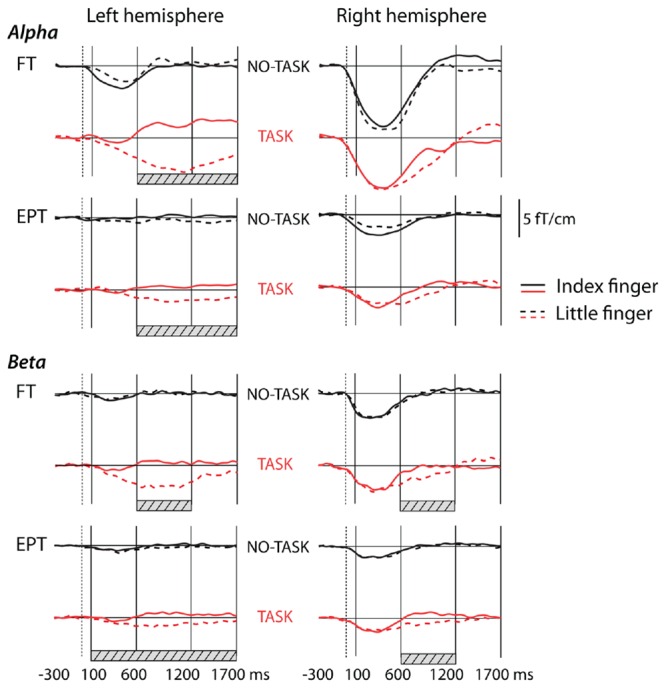
**Grand average curves showing modulation of oscillation amplitudes in alpha band and in beta band after tactile stimulus to the left hand in NO-TASK and TASK conditions in full term (FT, *n* = 21) and extremely preterm (EPT, *n* = 22) children.** The traces are grand averages of two channels in the left or right hemisphere over the sensorimotor cortices. The vertical solid lines indicate the time windows for analysis, the dotted lines show stimulus onset. The solid horizontal lines represent the prestimulus baseline level. Hatched bars show the time windows in which the curves for index and little fingers differed significantly (in the TASK condition).

In the NO-TASK condition, sensorimotor oscillation levels were suppressed after the tactile stimulation of both fingers in both FT and EPT children (**Figures 1** and **2**). Suppression of the oscillation amplitude was most prominent over the right, contralateral-to-stimulation, sensorimotor cortex, but clear modulation was also present over the ipsilateral hemisphere in some of the children. In the FT children, 3-way repeated ANOVA [*hemisphere* (left, right) × *time-window* (100–600 ms, 600–1200 ms, and 1200–1700 ms) × *finger* (index, little finger)] confirmed that the amplitudes during the first time window were lower in the right than in the left hemisphere [interaction of *hemisphere* × *time-window F*(1.2, 24.3) = 42.3; *p* < 0.001; *post hoc* for left (LH) vs. right hemisphere (RH) during the first time-window *F*(1, 20) = 42.5; *p* < 0.001], indicating that the relative suppression was more pronounced in the contralateral-to-stimulation hemisphere (**Figure 2**). Furthermore, the oscillation level was more suppressed during the first than second or third time-window [main effect of *time-window F*(1.2, 23.4) = 35.7; *p* < 0.001; *post hoc* contrasts: 100–600 ms vs. 600–1200 ms *F*(1, 20) = 32.2, *p* < 0.001, 100–600 ms vs. 1200–1700 ms *F*(1, 20) = 40.7, *p* < 0.001]. There were no differences in modulation amplitudes between the stimulated fingers [*F*(1, 20) = 0.007; *p* = 0.9]. These main features were also observed in the EPT group (**Figure 2**, EPT). Suppression was stronger in the contralateral, right hemisphere [interaction of *hemisphere* × *time-window F*(1.4, 29.8) = 14.9; *p* < 0.001; *post hoc* for LH vs. RH during the first time-window *F*(1, 21) = 14.7; *p* = 0.001], and the oscillation level was more suppressed during the first than second or third time-window [main effect of *time-window F*(1.4, 29.5) = 15.2; *p* < 0.001; *post hoc* contrasts 100–600 ms vs. 600–1200 ms *F*(1, 21) = 14.8, *p* = 0.002 or 1200–1700 ms *F*(1, 21) = 18.4, *p* < 0.001]. A slight tendency for a rebound of the oscillation amplitude during the last time-window was detected only for the index finger stimulation (*p* = 0.04, not corrected) and only for the FT children. No other condition or frequency band showed any rebound.

##### NO-TASK condition. Beta band

The sensorimotor beta-band oscillations in the NO-TASK condition behaved like the alpha-band oscillations. They were suppressed during the first time window, [main effect of *time-window* FT: *F*(1.3, 26.1) = 28.8; *p* < 0.001; EPT: *F*(1.4, 29.1) = 33.2; *p* < 0.001; *post hoc* contrast FT: 100–600 ms vs. 600–1200 ms *F*(1, 20) = 39.8, *p* < 0.001; 100–600 ms vs. 1200–1700 ms *F*(1, 20) = 28.1, *p* < 0.001; EPT: 100–600 ms vs. 600–1200 ms *F*(1, 21) = 41.9, *p* < 0.001; 100–600 ms vs. 1200–1700 ms *F*(1, 21) = 34.8, *p* < 0.001], the suppression being more pronounced in the contralateral-to-stimulation hemisphere (**Figure 2**) [interaction of *hemisphere* × *time-window* FT: *F*(1.4, 28) = 41, *p* < 0.001; EPT: *F*(1.6, 33.4) = 32.9, *p* < 0.001; *post hoc* for LH vs. RH during the first time-window FT: *F*(1, 20) = 42.8; *p* < 0.001; EPT: *F*(1, 21) = 15.5; *p* = 0.001].

##### TASK condition. Alpha band

The effect of the TASK was reflected in the modulation magnitude in the left, contralateral-to-squeeze hemisphere [Interaction of *hemisphere* × *time-window* × f*inger* FT: *F*(1.8, 35.4) = 19.5; *p* < 0.001; EPT: *F*(1.6, 33.7) = 9.8 *p* = 0.001, **Figure 2**]. In both groups, the Go TASK effect was seen as a prominent, long-lasting suppression for the target little finger stimulation during the second and third time-windows in the left hemisphere (index vs. little finger, FT: first time-window, mean diff (95% CI) = 4.7 (1.1–8.4), *p* = 0.08; second time-window: mean diff (95% CI) = 20.7 (14.9–26.5), *p* < 0.001; third time-window: mean diff (95% CI) = 21.7 (15.7–27.7), *p* < 0.001; EPT: first time-window, mean diff (95% CI) = 0.2 (-3.1–3.5), *p* = 1; second time-window: mean diff (95% CI) = 10.3 (4.8–15.7), *p* = 0.006; third time-window: mean diff (95% CI) = 11.1 (6.4–15.8), *p* < 0.001). In the right hemisphere, there were no significant differences between the fingers in any time-window in either group. The long-lasting suppression in the left hemisphere is a combination of the initial small suppression caused by the tactile stimulation of the ipsilateral hand, and of an overlaid suppression caused by the squeeze with the contralateral, right hand. The EMG-activity for the correct squeezes started on average (*n* = 19 FT + 17 EPT) at 325 ± 100 ms after the stimulus onset without significant differences between the groups.

##### TASK condition. Beta band

Also in the beta band, the combination of the target little finger stimulation and the following toy squeeze induced a long suppression in the left, contralateral-to-squeeze hemisphere [main effects of *time-window* FT: *F*(2, 40) = 33.4; *p* < 0.001; EPT: *F*(1.4, 28.6) = 21.3 *p* < 0.001, and *finger* FT: *F*(1, 20) = 10.3; *p* < 0.004; EPT: *F*(1, 21) = 42.2, *p* < 0.001; *post hoc t*-tests index vs. little finger in different time-windows and hemispheres; FT: LH 100–600 ms, mean diff (95% CI) = 3.1 (-3.1–9.4), *p* = 1; 600–1200 ms, mean diff (95% CI) = 15.8 (8.5–23), *p* = 0.001; 1200–1700 ms, mean diff (95% CI) = 7.8 (1.5–14.1), *p* = 0.1; EPT: LH 100–600 ms, mean diff (95% CI) = 3.4 (1–5.8), *p* = 0.04; 600–1200 ms, mean diff (95% CI) = 11.8 (8–15.7), *p* < 0.001; 1200–1700 ms, mean diff (95% CI) = 8.1 (3.9–12.2), *p* = 0.006]. A long suppression after the target little finger stimulation was also present in both groups in the right hemisphere, ipsilateral to the squeeze: during the middle time-window the oscillation level to the target index finger stimulation was significantly lower than the level to the non-target finger [FT: RH 100–600 ms, mean diff (95% CI) = 1.5 (-0.3–3.3), *p* = 0.6; 600–1200 ms, mean diff (95% CI) = 6.5 (3–10), *p* = 0.006; 1200–1700 ms, mean diff (95% CI) = -3.8 (-8.7–1.1), *p* = 0.7; EPT: RH 100–600 ms, mean diff (95% CI) = 1.4 (-0.9–3.8), *p* = 1; 600–1200 ms, mean diff (95% CI) = 8.7 (5.6–11.8), *p* < 0.001; 1200–1700 ms, mean diff (95% CI) = 3.2 (-1.3–7.7), *p* = 0.9].

##### TASK vs. NO-TASK comparison

**FIGURE 3:**
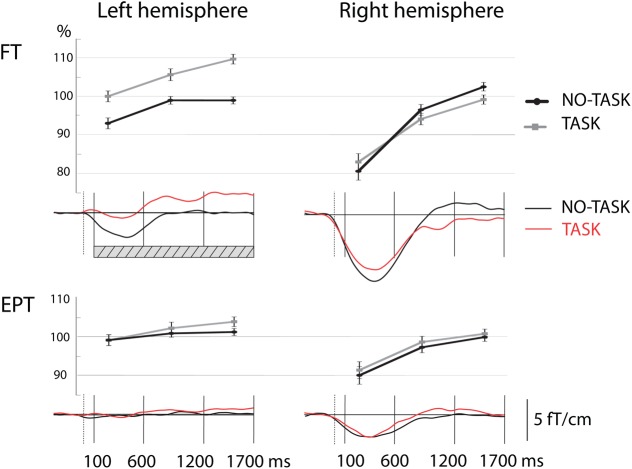
**Relative oscillation amplitude levels in the alpha band during the three time-windows in the left and right hemisphere after left index finger stimulation in NO-TASK and TASK conditions.** Line graphs (upper panels) show the mean relative values (±SEM) of oscillation level over the three time windows that were used for analysis. Lower panels show the modulation of oscillation level in time (grand average curves). Note the difference in oscillation levels of the FT group in the left hemisphere during TASK and NO-TASK conditions (time-windows with significant difference between the curves of the two conditions are shown by the hatched bar in the figure). FT, full term; EPT, extremely preterm.

To study the effect of the response inhibition, that is, the effect of TASK NoGo on post-stimulus alpha-oscillation levels, we made a comparison between the index finger stimulations in the TASK and NO-TASK conditions as the stimulus and behavioral response were the same in both conditions: index finger stimulation not followed by a toy squeeze (**Table 3**, **Figure 3**). In the FT children, the alpha band oscillation levels in the TASK NoGo condition, compared with the NO-TASK condition, were significantly higher during all time windows in the left, contralateral-to-omitted-squeeze, hemisphere. In the beta band a tendency for difference was present, but only in the latest time-window. In the EPT children, however, the oscillation levels did not differ between the two conditions during any time-window either in the alpha or beta band. In the right hemisphere, neither FT nor EPT children showed differences in any of the time-windows in any frequency band.

**Table 3:** Comparison of the levels of the oscillation amplitudes in the left hemisphere to index finger stimulation in TASK and NO-TASK conditions.

	FT				EPT			
Time-window	Alpha		Beta		Alpha		Beta	
	mean diff (95% CI)	*p*	mean diff (95% CI)	*p*	mean diff (95% CI)	*p*	mean diff (95% CI)	*p*
100–600 ms	6.9 (3.5–10.3)	0.003^∗^	0.9 (-1.3–3.2)	1	-0.02 (-4.0–4.0)	1	1.9 (-0.9–4.7)	1
600–1200 ms	6.8 (2.1–11.6)	0.04^∗^	1.7 (-1.2–4.5)	1	1.4 (-1.4–4.1)	1	3.3 (0.6–6)	0.1
1200–1700 ms	10.3 (6.9–13.6)	<0.001^∗^	2.7 (0.7–4.6)	0.06	2.7 (-0.3–5.6)	0.5	2.8 (-0.1–5.7)	0.3

*Two-tailed t-test, p-values are corrected for multiple comparisons, ^∗^p < 0.05; FT, full term; EPT, extremely preterm; CI, confidence interval.*

#### Source-Level Analysis

To avoid the effect of possible differences in the head position/size on the measured oscillation amplitudes, we did not use the sensor-level data in between-groups comparisons of sensorimotor oscillations. Instead, we performed this analysis on the source level, independent of the head position in the measurement helmet. First, equivalent current dipoles were calculated during the first prominent evoked-activity peak for index finger stimulation in the NO-TASK condition in the contralateral-to-stimulation, right hemisphere. Then, the alpha-band prestimulus oscillation level and relative suppression during the first time-window were calculated from the SI dipole source. The latency [FT: 45.6 ± 5.3 ms; EPT: 44 ± 5.4 ms (mean ± SD), mean diff (95% CI) = 1.6 (-1.7–5), *p* = 0.3] or strength [FT: 16.2 ± 11.8 nAm; EPT: 14.9 ± 8.9 nAm (median ± interquartile range), *p* = 1] of the equivalent SI dipole at the first major peak around 45 ms did not differ between the groups. Neither did the prestimulus alpha-band oscillation levels differ [FT: 11.3 ± 5.3 nAm; EPT: 11.5 ± 6.9 nAm (median ± interquartile range), *p* = 0.7]. However, the relative post-stimulus suppression during the first time-window was significantly stronger, and consequently oscillation level lower, in the FT than the EPT group [FT: 87.8 ± 9.7%, EPT: 93.6 ± 6.8% (mean ± SD); mean diff (95% CI) = 5.8 (0.3–11.2), *p* = 0.04, see also **Figure 2**].

#### Effect of Somatosensory TASK on Occipital Alpha Amplitude

**FIGURE 4:**
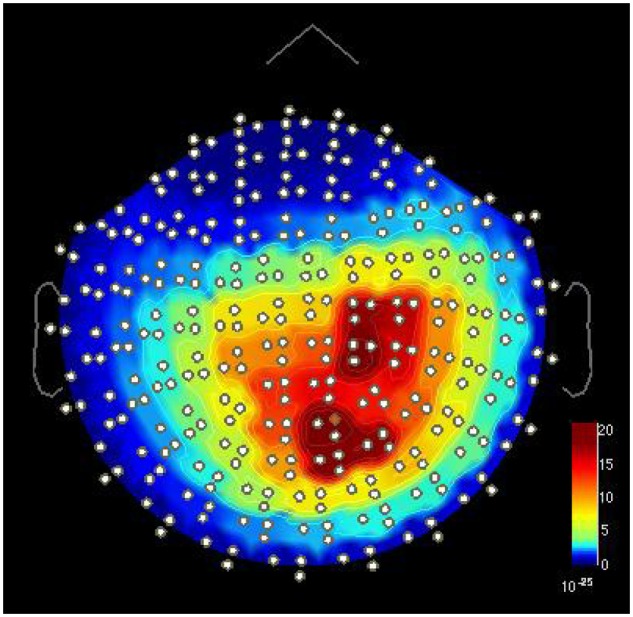
**Two separate alpha-band peaks around 9 Hz [power spectral density (T^2^/m^2∗^Hz) in MEG gradiometer channels] in one FT child in the TASK condition during the NoGo index finger stimulation.** One peak is located over the right sensorimotor area and the other over the occipital area. Visualized with Brainstorm software (Tadel et al., 2011).

In addition to the 9-Hz oscillations over the sensorimotor areas, spectral analysis revealed prominent alpha-band oscillations at 8.4 Hz over the occipital-midline area in both groups (**Figure 4**). The peak frequency difference between the occipital and sensorimotor alpha oscillations in the REST condition was significant in both groups (**Table 4**) with no between-group differences.

**Table 4:** Mean frequencies (±SD) of sensorimotor and occipital alpha oscillations in REST condition.

Frequency	FT (*n* = 21)	EPT (*n* = 21)	FT vs. EPT mean diff (95% CI)
Occipital alpha [Hz]	8.4 ± 0.8	8.4 ± 0.8	-0.002 (-0.5–0.5), *p* = 1
Sensorimotor alpha [Hz]	9.4 ± 1.0	9.1 ± 1.0	0.3 (-0.3–0.9), *p* = 0.6
Occipital vs. sensorimotor alpha mean diff (95% CI)	1.1 (0.5–1.6), *p* < 0.001^∗^	0.7 (0.4–1.1), *p* = 0.002^∗^	

*Two-tailed t-test, p-values are corrected for multiple comparisons, ^∗^p < 0.05; FT, full term; EPT, extremely preterm; CI, confidence interval.*

The effect of attending to somatosensory stimuli on occipital alpha levels was evaluated by comparing the relative occipital alpha levels (averaged over eight occipital channels) after the non-target index finger stimulation between the TASK (attention) and NO-TASK conditions. In the TASK condition, the level of the occipital alpha was significantly higher during the early time-window 100–600 ms (104.2 ± 4.9%; mean ± SD) compared with the corresponding time-window in the NO-TASK condition (100.5 ± 3.5%) with no group differences [interaction of *condition* (TASK vs. NO-TASK) × *time-window* (100–600, 600–1200, and 1200–1700 ms) *F*(2, 82) = 23.3 *p* < 0.001; *post hoc* for the early time-window, mean diff (95% CI) = 3.7 (1.9–5.5), *p* < 0.001]. During the intermediate time-window, the alpha levels between TASK and NO-TASK did not differ in the pooled data [TASK: 98.2 ± 6.1%; NO-TASK: 99.5 ± 4.1%; mean diff (95% CI) = -1.3 (-3.2–0.6), *p* = 0.5], and during the last time-window, the levels were reversed, i.e., the occipital alpha level was higher in the NO-TASK condition (TASK: 97.2 ± 4.9%; NO-TASK 99.5 ± 3.5%; mean diff (95% CI) = -2.3 (-3.8 to -0.9), *p* = 0.009).

## Discussion

We studied how 6-year-old children born EPT perform in comparison to FT children in a somatosensory Go/NoGo task. Furthermore, using MEG, we measured the reactivity of brain oscillations during this Go/NoGo (TASK) condition, and during a condition with the same tactile stimuli when the children were not paying attention nor responding to the stimuli (NO-TASK). Our data show that not only did the EPT children perform significantly worse than their FT born peers in the Go/NoGo task, but the reactivity of their sensorimotor network brain oscillations significantly differed from that of the FT children. Most importantly, sensorimotor oscillations were enhanced during response inhibition in the FT children but not in the EPT children. We suggest that the observed differences in neural oscillations during the NoGo task reflect altered brain mechanisms employed for response inhibition in the children born EPT.

### The NO-TASK Condition — Effect of Stimulus on the Oscillation Levels

Tactile stimulation of the fingers induced, expectedly, a prominent suppression of the sensorimotor oscillations on the contralateral-to-stimulation hemisphere within the first time-window. This suppression was, however, significantly weaker in the EPT than FT children, which may reflect reduced ability to engage the stimulus-relevant brain networks into active information processing. In a recent experiment, a group of autistic children showed reduced posterior alpha-band suppression in comparison with typically developing children while pantomiming tool use (Ewen et al., 2016). Decreased modulation of alpha oscillation was interpreted to indicate decreased task-related activation.

In adults, the post-stimulus suppression is followed by a rebound of the oscillations over the baseline levels. In the present data set, some individual traces showed such a rebound, but it did not reach significance in the group-averaged data either in FT or EPT children, alpha or beta band. A previous study (Gaetz et al., 2010) suggests that at least post-movement beta rebound, thought to reflect motor inhibition, gradually develops with age, and 4–6-year-olds still show significantly reduced post-movement beta rebound in comparison to older groups. It is thus possible that the stimulus-induced rebound also develops with age and was therefore not well seen in our 6-year-old subjects.

### The Go/NoGo TASK Condition — Behavioral Performance and Its Oscillatory Correlates

In the TASK condition, the EPT children responded less often to the target stimulation, as well as more often to the non-target stimulation than the FT children. In addition, their performance was even less precise for the non-target than target finger, indicating that it was more difficult for them to refrain from responding to the non-target index finger stimulation than to respond to the target little finger stimulation. These results are in line with those of others who have studied response inhibition in preterm children with a Go/NoGo task (Mulder et al., 2011; Orchinik et al., 2011; Baron et al., 2012; Loe et al., 2015).

The poorer performance of the EPT children in the NoGo TASK (requiring response inhibition) was reflected in the modulation of sensorimotor brain oscillation levels. In FT children, in the left hemisphere—contralateral to the hand squeezing the toy—the relative sensorimotor alpha oscillation level was higher in the NoGo TASK compared with the NO-TASK condition. In other words, the oscillation level was enhanced when the FT children were actively refraining from squeezing, in contrast to when they were not paying attention to the stimulation, even though the behavioral response—no squeeze—was the same in both conditions. The EPT children, interestingly, showed no such difference between the NoGo TASK and NO-TASK conditions. We suggest that the enhanced sensorimotor oscillations in the left hemisphere when refraining from squeezing (i.e., NoGo) reflect active motor response inhibition. The lack of this effect in EPT children seems to therefore reflect altered brain mechanisms employed to actively inhibit a motor response.

In the Go TASK condition, the toy-squeeze induced a remarkable suppression on the contralateral-to-squeeze hemisphere in both EPT and FT children with a peak during the middle time-window. The peak latency of this long-lasting suppression is evidently a consequence of the reaction time after the stimulation was felt, as the squeeze started around 325 ms after the stimulation. Although, by visual inspection, the squeeze-induced suppression in the left hemisphere seemed stronger in the FT children, the suppression was also significantly present in the EPT children. As we could not adequately quantify the motor activity of the squeeze, we did not compare the suppression in the Go TASK condition between FT and EPT children, since group differences in the squeeze itself could have as well explained differences in the oscillation levels.

In a previous somatosensory Go/NoGo study in adults, a NoGo condition induced a prominent increase in alpha (and beta) level in frontal and central areas (pooled data from several sensors over both hemispheres; Nakata et al., 2013). In our study, even in the FT children the oscillation level was elevated only slightly, and merely on the contralateral-to-squeeze hemisphere. This alpha-increase in the NoGo TASK condition was seen by visual evaluation in individual subjects mostly on channels contralateral to the omitted squeeze over the central or fronto-central areas. One explanation for the more local/moderate elevation levels in our young subjects might be that, as response inhibition in a Go/NoGo task is known to develop with age (Levin et al., 1991; Brocki and Bohlin, 2004), its cortical expression reflected in elevated oscillation levels may simply not yet be fully developed in 6-year-old children. As with the EPT children, they did not show this elevated alpha level at all. However, two cross-sectional research reports suggested that, in response inhibition task performance, very preterm children approaching adolescence may catch up with their FT peers (Aarnoudse-Moens et al., 2012; Ritter et al., 2013). Whether the lack of sensorimotor oscillation enhancement during response inhibition in our group of 6-year-old EPT children would be a delay rather than a deficit remains a prospect for future research.

### Neural Substrates of Response Inhibition

Neural substrates of response inhibition have been widely studied with fMRI. These studies have revealed a wide cortical network underlying response inhibition (for reviews, see e.g., Simmonds et al., 2008; Swick et al., 2011; Criaud and Boulinguez, 2013). This wide activation may, however, be at least partly explained by non-specific paradigms also activating several other cognitive processes that are intrinsically related to response inhibition and difficult to disentangle, including, e.g., attention, working memory, and response selection (Criaud and Boulinguez, 2013). FMRI studies of response inhibition in preterm-born adolescents/adults have shown differences in BOLD responses in several brain areas (but not the sensorimotor areas) in the absence of behavioral differences (Nosarti et al., 2006; Lawrence et al., 2009). The brain activation differences were suggested to represent altered neuronal pathways for response inhibition in the preterm-born group. In another study, preterm children (6–7 years) with intrauterine growth restriction exhibited higher variability in executing or inhibiting a response than preterm-born children without growth restriction and also ‘decreased reliance on brain regions classically involved in successful response inhibition’ (Réveillon et al., 2013).

The lack of activation/deactivation of sensorimotor cortices in fMRI studies of response inhibition is striking. Nakata et al. (2008) reported activation of some sensorimotor areas in addition to several other brain areas in NoGo trials, requiring inhibition of movement after electric stimulation of hand nerves. However, the activation was not contrasted with the same stimulation in a no-task condition, so it remains unknown if it was enhanced during response inhibition. More time-sensitive methods, however, do show changes in sensorimotor-cortex activity. In an MEG study on response inhibition, the NoGo stimulus started to activate the motor cortex 100–120 ms after the stimulus onset, just like the Go-stimulus, with a consequent decay of activity before 200 ms, indicating initial preparation for and subsequent inhibition of the movement (Endo et al., 1999). In a transcranial magnetic stimulation (TMS) study, motor evoked potentials (MEPs) were suppressed during a NoGo condition, reflecting inhibition in the pyramidal tract (Hoshiyama et al., 1996). Furthermore, behavioral and electrophysiological (decreased MEP amplitudes) response inhibition was shown to be paralleled by enhanced oscillatory alpha activity over cortical sensorimotor areas (Hummel et al., 2002). It is thus plausible that the elevated oscillation levels in our study reflect inhibition of motor output.

### Cross-Modal Influences – Effects of Somatosensory Stimuli and TASK on Visual Alpha Oscillations

Two separate spectral peaks in the alpha range were detected in spectral analyses in all conditions, one over the occipital and the other over sensorimotor areas. In both EPT and FT children, the peak frequency (in the REST condition) of the sensorimotor alpha (∼9.3 Hz) was slightly but significantly higher than the occipital alpha (∼8.4 Hz). This is in agreement with the slightly higher sensorimotor than occipital alpha frequencies reported in adults (Storm van Leeuwen et al., 1978). In our study, the alpha frequencies in the sensorimotor or occipital areas did not differ between FT and EPT children. In other brain areas, differences have been shown by Doesburg et al. (2011): compared with full-term children, preterm born children (≤32 week of gestation) had lower peak alpha frequencies in frontal areas and lower alpha power in bilateral frontal and temporal areas.

In the present study, the level of the occipital alpha was significantly enhanced during the first time-window in the NoGo TASK condition, while attending to the somatosensory stimuli, compared with the occipital alpha level with the same index-finger stimulation in the NO-TASK condition when the stimuli were not attended. Several studies suggest that both in visual and somatosensory modalities, cue-induced alpha modulation might serve as a gating mechanism for attention. Alpha band oscillations have been suggested to reflect frontally controlled disengagement of task-irrelevant regions (Haegens et al., 2010). For example, cued visual spatial attention leads to increased amplitude of occipital oscillatory alpha activity in the hemisphere contralateral to the unattended visual field (Worden et al., 2000). The same holds for the somatosensory area: attention increased the power of sensorimotor alpha and beta oscillations in the hand area of SI when attention was directed to the foot, and decreased the power when attention was cued to the hand (Jones et al., 2010). This reciprocal effect is also intermodal: visual processing increases the sensorimotor alpha and decreases the visual alpha, and movement or somatosensory attention decreases the sensorimotor alpha and increases the visual alpha (Pfurtscheller, 1992; Anderson and Ding, 2011). A similar intermodal effect was seen in the early time-window in our study – when the children were attending to the somatosensory stimuli, the visual-alpha levels were enhanced.

The relative sensorimotor oscillation level associated with the response inhibition in the FT group was higher in the TASK NoGo compared with the same finger stimulation in the NO-TASK condition. Also, the relative occipital alpha levels in the occipital channels were higher in the TASK NoGo compared with the NO-TASK condition. The natural doubt arises whether this response inhibition effect is a ‘leakage’ of the occipital alpha to the sensorimotor areas. The occipital alpha level was, however, significantly higher in the TASK than NO-TASK condition only during the first time-window, whereas the sensorimotor alpha level continued to be higher in the TASK than NO-TASK during the middle and last time-windows. Thus, the occipital alpha cannot explain the enhancement of the oscillation level in the sensorimotor channels, and the response inhibition effect seen as enhanced sensorimotor alpha levels during the TASK NoGo vs. NO-TASK condition is, therefore, genuine.

### Possible Limitations of the Study

One of the methodological limitations of the study is that the main analysis was carried out on the sensor level. The sensor level analysis should not have affected most of our results, as they were gained from comparisons that were based on relative changes within the study groups. Results of between-group comparisons could have been affected by, e.g., different head shapes and consequently different distances of relevant cortex from the sensors, resulting in smaller amplitude of the measured oscillation. For between-groups comparisons, we therefore calculated the prestimulus oscillation level and relative suppression from the SI dipole source waveform. As the equivalent dipole source calculation takes into account the head position and distance of the measured activity from the sensors, we obtained oscillation values from the SI dipoles independent from the head size/form. The post-stimulus suppression, measured from the SI source waveform data, was indeed weaker in the EPT than the FT children in these comparisons.

In addition, the last nine FT children were recorded with an upgraded MEG-device (TRIUX), whereas all EPT children were recorded with the previous device (Vectorview). We do not believe the change in the device had significant impact on our results, however, as a preliminary analysis including only the FT children (*n* = 14) measured with the Vectorview-device yielded the same main results (Pihko et al., 2015).

In this study, we excluded children with major abnormalities in brain imaging because these lesions might have affected the MEG results. Subsequently, none of the EPT children in this study had cerebral palsy or severe cognitive impairment. Excluding the most severely affected patients from our study resulted presumably in better behavioral performance and possibly less altered MEG results among the EPT children.

The number of subjects—22 EPT children and 21 FT children—was limited, but sufficient to detect at least the most robust differences. Since the relatively small sample size of the subject groups, we used the most conservative correction for multiple comparisons (Bonferroni) to bring out merely the most clinically relevant results. Confidence intervals further support these results and our conclusions.

## Conclusion

Our data show that the reactivity of the sensorimotor oscillations differed between the EPT and FT children in at least two ways. First, the stimulus-induced suppression in the hemisphere contralateral to the stimulated hand was weaker in the EPT children. Secondly, the response inhibition in the TASK NoGo was associated with elevated oscillation level in the hemisphere contralateral to the hand refraining from squeezing in the FT, but not in the EPT children. Taken together, these differences suggest that in EPT children, even in the absence of major brain abnormalities, the sensorimotor network functions differ from peers born FT. It will be interesting to see in the future studies whether these differences remain in adolescence, or if the EPT children will catch up with their full-term peers both behaviorally and in brain measures of sensorimotor network function.

## Ethics Statement

The Ethics Committee for gynaecology and obstetrics, pediatrics and psychiatry of the Hospital District of Helsinki and Uusimaa approved the study protocol. All participating children and their parents gave written informed consent before participation in accordance with the Declaration of Helsinki. All the performed examinations in this study are considered harmless to the participants. All children were given age-appropriate information about the study prior to participating.

## Author Contributions

EP, PL, and PN contributed to study concept and design, acquisition and analysis of the data, and drafting the initial manuscript. LL contributed to study concept and design, and data analysis. AL, MM, and SA contributed to the clinical aspects of the study. EW was responsible for the cognitive ability testing. All authors contributed to the interpretation of the results, revising the manuscript for important intellectual content, and have read and approved the final version of the manuscript.

## Conflict of Interest Statement

The authors declare that the research was conducted in the absence of any commercial or financial relationships that could be construed as a potential conflict of interest.
